# Effect of extensive mesenteric excision on primary ileocolic resection outcomes in Crohn’s disease patients: a systematic review with meta-analysis

**DOI:** 10.1007/s00384-025-05043-0

**Published:** 2025-12-02

**Authors:** Aleix Martínez-Pérez, Carlo Alberto Schena, Gianluca Pellino, Elías Martínez-López, Danila Azzolina, Nicola de’Angelis

**Affiliations:** 1 Unit of Colorectal Surgery, Department of General and Digestive Surgery, Hospital Universitario Doctor Peset, Valencia, Spain; 2 Department of Surgery, University of Valencia, Valencia, Spain; 3 General Surgery Department, Fondazione Poliambulanza, Brescia, Italy; 4 Department of Translational Medicine and LTTA Centre, University of Ferrara, Ferrara, Italy; 5 Colorectal Unit, Department of General and Digestive Surgery, Vall d’Hebron University Hospital, Universitat Autonoma de Barcelona UAB, Barcelona, Spain; 6 Department of Advanced Medical and Surgical Sciences, Università Degli Studi Della Campania Luigi Vanvitelli, Naples, Italy; 7 Department of Environmental and Preventive Science, University of Ferrara, Ferrara, Italy

**Keywords:** Crohn’s Disease, Mesenterium, Ileocolic resection

## Abstract

**Purpose:**

The role of mesenteric excision in Crohn’s Disease (CD) remains uncertain. We aimed to evaluate the impact of extended vs. limited mesenteric excisions on intra- and postoperative outcomes in patients undergoing primary ileocolic resection for CD.

**Methods:**

A systematic search was conducted in PubMed, EMBASE, Web of Science, ClinicalTrials.gov, and ISRCTN up to February 2025. Randomized controlled trials (RCTs), non-RCTs, and retrospective studies comparing extended and limited mesenteric excision in primary ileocolic resections for CD were included. The primary outcome was endoscopic CD recurrence (Rutgeerts score ≥ i2). Secondary outcomes included severe endoscopic recurrence (≥ i2b or ≥ i3), surgical recurrence, anastomotic leaks, operative time, conversion to open surgery, severe postoperative complications, and length of hospital stay.

**Results:**

Over the 2588 records initially screened, 4 studies were included, involving a total of 632 patients. Pooled analysis showed no significant difference in endoscopic recurrence rates between extended and limited resections (48.2% vs. 54.1%; RR: 0.91; 95% CI: 0.70–1.18; p = 0.46; I^2^ = 57%). Additionally, there were no significant differences in the risk of anastomotic leak (3.8% vs. 2.6%; RR: 1.35; 95% CI: 0.14–12.88; p = 0.80; I^2^ = 52%) or any other analyzed outcomes.

**Conclusion:**

Extended mesenteric excision does not appear to significantly reduce endoscopic recurrence compared with limited excision in primary ileocolic resections for CD. Until further high-quality evidence is available, surgical teams should adhere to their established practice and refrain from implementing extended resections outside well-designed prospective studies.

**Registration:**

PROSPERO (CRD42025644791).

**Supplementary Information:**

The online version contains supplementary material available at 10.1007/s00384-025-05043-0.

## Introduction

Crohn’s disease (CD) is a chronic, relapsing inflammatory disorder of the gastrointestinal tract, with an estimated prevalence of 0.3% in Western countries and a rising incidence in developing and newly industrialized nations [1, 2]. The burden of CD extends beyond its medical implications, significantly affecting healthcare systems, patients, and society. The disease often manifests during early adulthood, leading to prolonged healthcare utilization, productivity loss, and a profound impact on patients' quality of life [3, 4].

Despite advancements in medical therapy, surgery remains a cornerstone in the management of ileocolic CD [5]. Upfront surgical resection has been associated with lower relapse rates and reduced need for maintenance biologic therapy compared with initial medical treatment, making it a reasonable and cost-effective alternative, particularly in biologic-resistant populations [6]. Primary ileocolic resection for CD demonstrates excellent perioperative outcomes in experienced hands, with fewer than 5% of patients experiencing severe postoperative complications and only 3% developing anastomotic leaks [7]. Furthermore, the use of laparoscopy for CD is associated with reduced rates of incisional hernia and obstruction, and with improved cosmesis [8]. However, the impact of preoperative biologic exposure on postoperative outcomes remains a topic of debate [9].

Historically regarded as a passive anatomical structure, the mesentery has recently been recognized as a key player in the pathogenesis and recurrence of CD [10, 11]. Emerging evidence suggests a role for the mesentery, and especially the presence of creeping fat (a hallmark of CD), in chronic inflammation, fibrosis, and immune activation [12]. Retrospective studies have indicated that extended mesenteric excision (EME) may lower surgical recurrence rates compared with conventional limited mesenteric excision (LME) in ileocolic resection [13]. EME involves the resection of the affected mesenteric tissue along with the diseased bowel segment, aiming to remove all sources of chronic inflammation. Given these findings, EME has been proposed as a surgical strategy to reduce postoperative recurrence in CD patients. However, concerns remain regarding whether EME increases surgical complexity or impairs blood supply to the ileocolic anastomosis, thereby raising the risk of anastomotic leak. The most recent guidelines concluded that there is insufficient evidence to recommend EME in surgery for ileocolic CD [5]. Recent meta-analysis based on studies including different types of intestinal resections for CD have sparked controversy, suggesting either a benefit of EME in reducing surgical recurrence [14, 15] or no benefit [16, 17].

To address these issues, the present systematic review synthesizes the available evidence and assesses the impact of EME in primary ileocolic resection for CD, focusing on whether it is associated with reduced recurrence rates without increasing perioperative adverse events.

## Methods

### Study design

The study methods were documented in a protocol developed a priori in accordance with current standards and were prospectively registered on the International Prospective Register of Ongoing Systematic Reviews (PROSPERO) under registration number CRD42025644791. The Preferred Reporting Items for Systematic Reviews and Meta-Analysis (PRISMA) statement checklist was followed [18] (eTable 1). The present work is also AMSTAR-2 compliant [19] (eTable 2).

### Selection criteria

The primary aim of the present study was to compare the effectiveness of EME vs. conventional LME for primary ileocolic resection in CD. LME was defined as a mesenteric division close to the bowel, whereas EME was defined as extensive mesenteric resection near the level of the ileocolic trunk.

The eligibility and selection criteria were defined before initiating the literature search to ensure the proper identification of all studies to be included in the systematic review. Randomized controlled trials (RCTs), prospective non-RCTs, and retrospective studies addressing the review question were retrieved and analyzed. Studies comparing EME vs. LME for CD, including other intestinal locations, were considered for inclusion if separate results for the ileocolic resection subgroup were provided. Papers reporting case series, colorectal cancer, or other benign entities were excluded.

By applying the PICOTS framework, the study selection criteria were established as follows:

- *(P) Participants*: Adult patients requiring primary ileocolic resection for CD.
- *(I) Intervention*: EME.
- *(C) Comparison:* Conventional LME.
- *(O) Outcome measures*:
  - The primary outcome was the percentage of endoscopic recurrence, defined by the finding of a Rutgeerts scale ≥ i2 in endoscopic surveillance [20].
  - Secondary outcomes included: a) severity of endoscopic recurrence (Rutgeerts ≥ i2b or ≥ i3, %); b) surgical recurrence (%), defined as the need for further surgical intervention due to disease recurrence in the ileocecal area; c) operative time (min); d) conversion to open surgery (%); e) anastomotic leak (%); f) severe postoperative complications (%); e) length of hospital stay (days).

- (T) Time: Short- and long-term.
- (S) Setting: Inpatient and outpatient.

### Literature search strategy

A literature search was primarily performed in the following online databases and electronic trial registries: PubMed, EMBASE, Web of Science, www.clinicaltrials.gov, and www.isrctn.com. Comprehensive literature searches were performed from inception to 2 February 2025. To increase the odds of identifying all relevant articles, queries were adapted to each database. Appropriate limits were set using combinations of Boolean operators, controlled vocabulary terms, keywords, natural language, or truncated words (eTable 3). These terms were related to the PICOTS research question (e.g., inflammatory bowel disease, Crohn’s disease, mesentery, surgical procedures, recurrence). No restrictions were applied for language or publication year. Non-English papers were translated. Grey literature was searched through Google Scholar and databases such as the Grey Literature Report or OpenGrey. Reference lists of eligible studies were hand-searched to identify additional publications. The last literature search was performed on 30 July 2025. When available, the ‘‘related articles’’ function was used to broaden the search. All identified records were exported to the citation management tool EndNote X20 (Clarivate Analytics, Philadelphia, PA, USA) for reference deduplication.

### Study selection

Following deduplication, the titles and abstracts of the retrieved articles were independently and blindly screened for relevance by two reviewers (AMP, CAS). To maximize sensitivity, records were removed only if both reviewers excluded them at the title/abstract screening level. Subsequently, both reviewers performed a full-text analysis of the remaining articles, after which the final selection was made. Disagreements were discussed with a third researcher (NdA) until a consensus was reached.

### Data extraction

The data initially sought were as follows: (1) general study information (e.g., year of publication, country); (2) participant characteristics (e.g., age, disease duration, Montreal classification, smoking status, use of medications) and inclusion/exclusion criteria; (3) intervention details (e.g., procedure, approach, type of anastomosis), (4) long-term follow-up, and (5) funding. Outcome measures were independently extracted for each surgical treatment by the reviewers (AMP, CS). Discrepancies were resolved by consensus with a third researcher (NdA). When possible, outcome variables were calculated based on the data provided in the individual selected studies. When necessary, the authors of the original studies were contacted to clarify the results or to provide updates or corrections. All data were recorded in a dedicated database. Binary data were collected as the total number of events and patients at risk. To summarize continuous outcomes in studies in which the mean or SD were not reported, these values were estimated from median, ranges, interquartile ranges (IQR), or *p* values [21].

### Quality assessment

Methodological quality and compliance with the corresponding standard of outcome reporting for each study (i.e., RCT, prospective NRCT, and retrospective comparative studies) were assessed by two independent researchers. For RCTs, reviewers evaluated the risk of bias using the Cochrane “Risk of Bias 2” (RoB 2) assessment tool [22]. For NRCTs and retrospective studies, reviewers evaluated the risk of bias using the Cochrane ROBINS-I V2 tool [23]. These tools require the assessment of specific variables across several domains. Each domain is judged as low or high risk, or ‘some concerns’, leading to an overall assessment as low risk, high risk, or ‘some concerns’. Risk-of-bias plots were generated using the Robvis App [24]. The quality of evidence was assessed using the Grading of Recommendations Assessment, Development, and Evaluation (GRADE) system [25]. Funnel plots were prepared to evaluate publication bias in all analyses.

### Pooled data analysis

Data were processed using qualitative and quantitative analyses. Descriptive synthesis was used to summarize the study and patient characteristics, details of the different interventions, surveillance work-up, and risk-of-bias evaluation. For binary outcome variables, the relative risk (RR) and 95%CI were estimated using the Mantel–Haenszel method; an RR < 1 favored EME. For continuous outcomes, the weighted mean differences (MD) and 95% CI were estimated using inverse-variance weighting; a negative MD favored EME. The pooled effect was considered significant if p < 0.05.

Due to the expected interstudy clinical heterogeneity, a random effects model was used to calculate the pooled estimates of treatment efficacy to adopt a more conservative approach. All analyses were performed according to the intention-to-treat principle, except those assessing endoscopic recurrence, which were conducted on a per-protocol basis (i.e., patients undergoing endoscopic surveillance). Statistical heterogeneity was assessed by the I^2^ statistic [26–28]. I^2^ values of 25%, 50%, and 75% were considered to indicate low, moderate, high, or very high heterogeneity, respectively [27, 28]. If at least three RCTs were available, subgroup analyses including only RCTs were planned.

## Results

### Literature search and selection

**Fig. 1:**
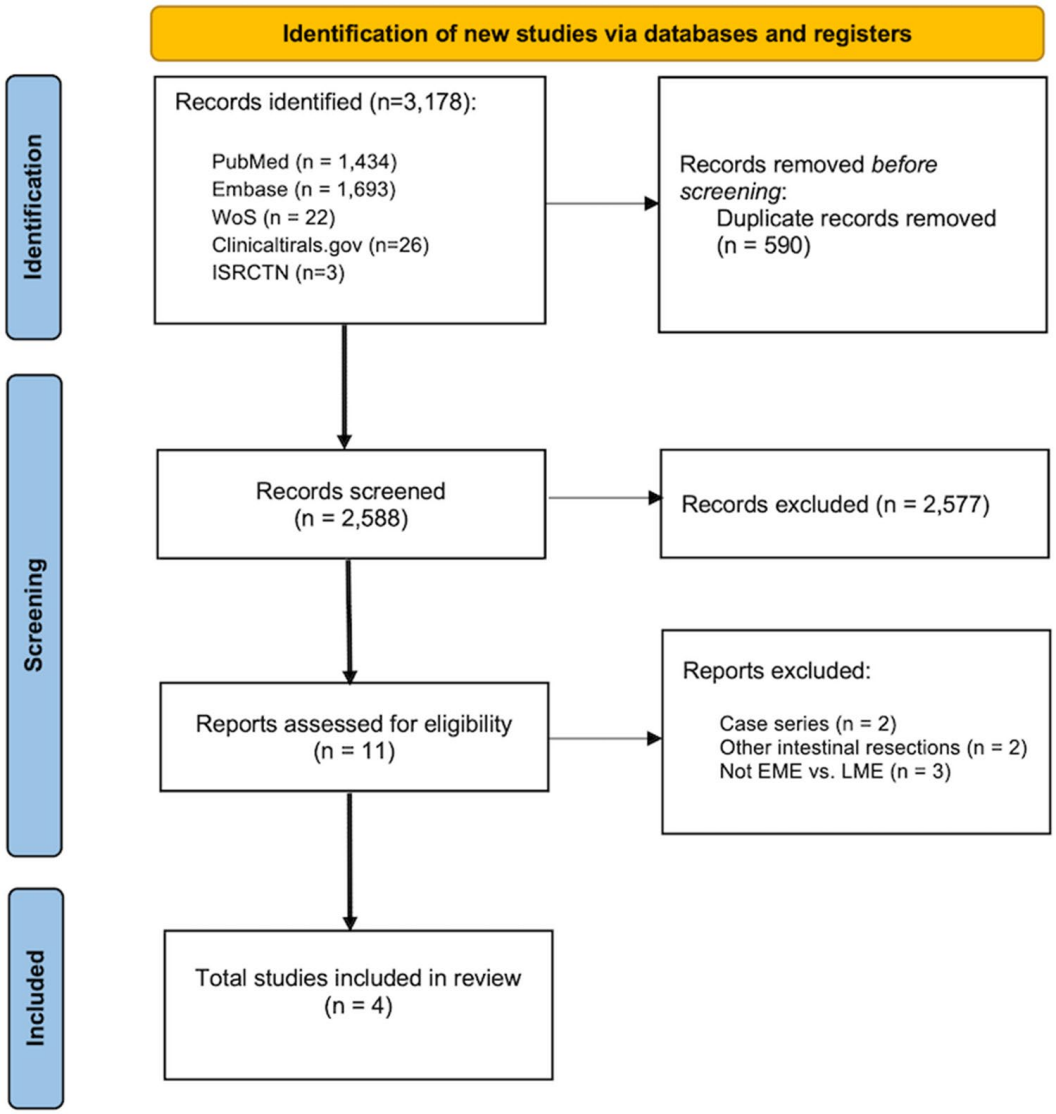
PRISMA 2020 flow diagram

The comprehensive literature search identified a total of 3,178 articles. After removing duplicates (n = 590), 2,577 studies were excluded by screening titles and abstracts. The remaining 11 records underwent full-text analysis, and 4 studies fulfilled the inclusion criteria. No additional studies meeting the inclusion criteria were identified through Google Scholar and OpenGrey searches. The flowchart of the literature search and study selection process is shown in Fig. 1. The list of studies screened at full-text analysis and the reasons for exclusion are reported in eTable 4. Nine registered unpublished studies were also found.

### Study characteristics

The included studies were published between 2018 and 2025. Two were multicenter RCTs [29, 30] and the other two were mono- [13, 31] or bicenter [32] retrospective studies. They included patients with CD who underwent surgery between January 2000 and April 2023. Overall, these studies analyzed a total of 632 patients undergoing primary EME or LME for ileocolic CD. There were 362 patients (43.9% female) in the EME group and 270 patients (43.7% female) in the LME group. The main characteristics of the included studies are presented in Tables 1 and 2.

**Table 1:** Summary of the included studies

				Smoker n (%) (active/former/never)		Age at onset (A1/A2/A3)		Behavior of disease (B1/B2/B3)		Location of the disease		Perianal disease		Medications before surgery	
Study (year)	Country (centres)	Study period	n (EME/LME)	ER	LR	ER	LR	ER	LR	ER	LR	ER	LR	ER	LR
van der Does de Willebois et al (2024) [26]	Netherl. Italy (6)	Feb 2020 Apr 2023	131 (66/65)	15 (22%) 15 (22%) 37 (55%)	16 (24%) 9 (14%) 41 (62%)	4 (6%) 38 (57%) 25 (37%)	8 (12%) 40 (61%) 18 (27%)	22 (33%) 28 (42%) 17 (25%)	15 (23%) 29 (44%) 22 (33%)	L1 44 (66%) L3 23 (34%)	L1 40 (61%) L3 26 (39%)	5 (7%)	12 (18%)	None 24 (36%) Mesalazine 0 Thiopurines 8 (12%) Biologics 35 (52%) Small molecules 0	None 28 (42%) Mesalazine 1 (2%) Thiopurines 10 (15%) Biologics 25 (38%) Small molecules 2 (3%)
Duan et al (2025) [27]	China (3)	Feb 2019 Dec 2022	111 (58/53)	2 (3%) 7 (12%) 49 (85%)	2 (4%) 1 (2% (94%)	n.r	n.r	0 (0%) 29 (50%) 29 (50%)	0 (0%) 30 (57%) 23 (43%)	L1/L3	L1/L3	17 (30%)	19 (36%)	None 39 (67%) Immunomodulators 8 (14%) Steroids 2 (3%) Biologic 9 (16%)	None 35 (66%) Immunomodulators 9 (17%) Steroids 0 Biologic 9 (17%)
Mineccia et al (2022) [28]	Italy (2)	Jan 2009 Dec 2019	326 (204/122)	75 (37%)* 129 (63%)*	35 (29%)* 87 (71%)*	12 (6%) 140 (69%) 52 (25%)	15 (12.3%) 68 (55.7%) 39 (32%)	0 (0%) 67 (33%) 137 (67%)	0 (0%) 40 (33%) 82 (67%)	L1?	L1?	27 (13%)	16 (13%)	Washout/5-ASA 114 (55.9%) Steroids 28 (13.7%) Immunosuppressants 21 (10.3%) Biologicals 32 (15.7%) Combined therapy 9 (4.4%)	Washout/5-ASA 46 (38%) Steroids 34 (28%) Immunosuppressants 13 (10%) Biologicals 16 (13%) Combined therapy 13 (11%)
Coffey et al (2018) [13]	Ireland (1)	Jan 2004 unknown	64 (34/30)	18 (53%) 2 (6%) 13 (38%)	14 (47%) 6 (20%) 9 (30%)	26 (76%)** 6 (18%)**	23 (77%)** 6 (20%)**	8 (24%) 14 (41%) 12 (35%)	16 (53%) 6 (20%) 8 (27%)	L1 6 (76%) L2 0 (0%) L3 6 (18%) L4 2 (6%)	L1 23 (77%) L2 2 (6%) L3 5 (17%) L4 0 (0%)	n.r	n.r	Anti-inflammatory 9 (27%) Steroid 12 (35%) Immunosuppressant 10 (29%) Biologic15 (44%) None 5 (15%) Data unavailable 2 (6%)	Anti-inflammatory 15 (50%) Steroid 13 (43%) Immunosuppressant 11 (37%) Biologic 5 (17%) None 5 (17%) Data unavailable 1 (3%)

^*^ smoker yes/no; ** Vienna Classification A1/A2

EME stands for Extensive Mesenteric Excision, LME stands for Limited Mesenteric Excision

**Table 2:** Characteristics of the included randomized controlled trials

TRIAL	Control arm (LME)	Experimental arm (EME)	Design	Primary outcome	Post-randomisation drop-outs	Laparoscopy n (%)	Type of anastomosis n (%)
SPICY trial ^26^	The mesentery was divided close to the bowel	Resection up to the level of the ileocolic trunk without ileocolic pedicle section	Superiority	6-month postoperative endoscopic recurrence rate (Rutgeerts ≥ i2b)	ER = 5 - 4 protocol violation (1 no anastomosis, 2 diagnosis other than Crohn's disease postoperatively, 1 withdrew informed consent) - 1 protocol deviation (1 lost to follow-up) LR = 3 - 2 protocol violation (1 no anastomosis created, 1 withdrew informed consent) - 1 protocol deviation (1 lost to follow-up)	EME: 67 (100%) LME: 66 (100%)	EME: 66 (99%) stapled, 46 (69% anisoperistaltic) LME: 66 (100%) stapled, 41 (62% anisoperistaltic)
MESOCOLIC trial ^27^	The mesentery was retained, i.e., “close shave” or < 3 cm from the border of bowel	Resection following CME, avoiding the root region, (i.e., 1 cm from the root)	Superiority	Surgical recurrence: Repeat surgery for a CD-related indication caused by recurrent disease in the same anatomical areas	No P-R dropouts as all 111 randomized patients underwent operation, but in 26 (23.4%) there was no endoscopic assessment (drop-out only for the interim analysis)	EME: 39 (74%) LME: 41 (71%)	EME: 58 stapled, Isoperistaltic 34 (59%) LME: 53 stapled (100%), Isoperistaltic 37 (70%)

### Primary outcome

**Fig. 2:**
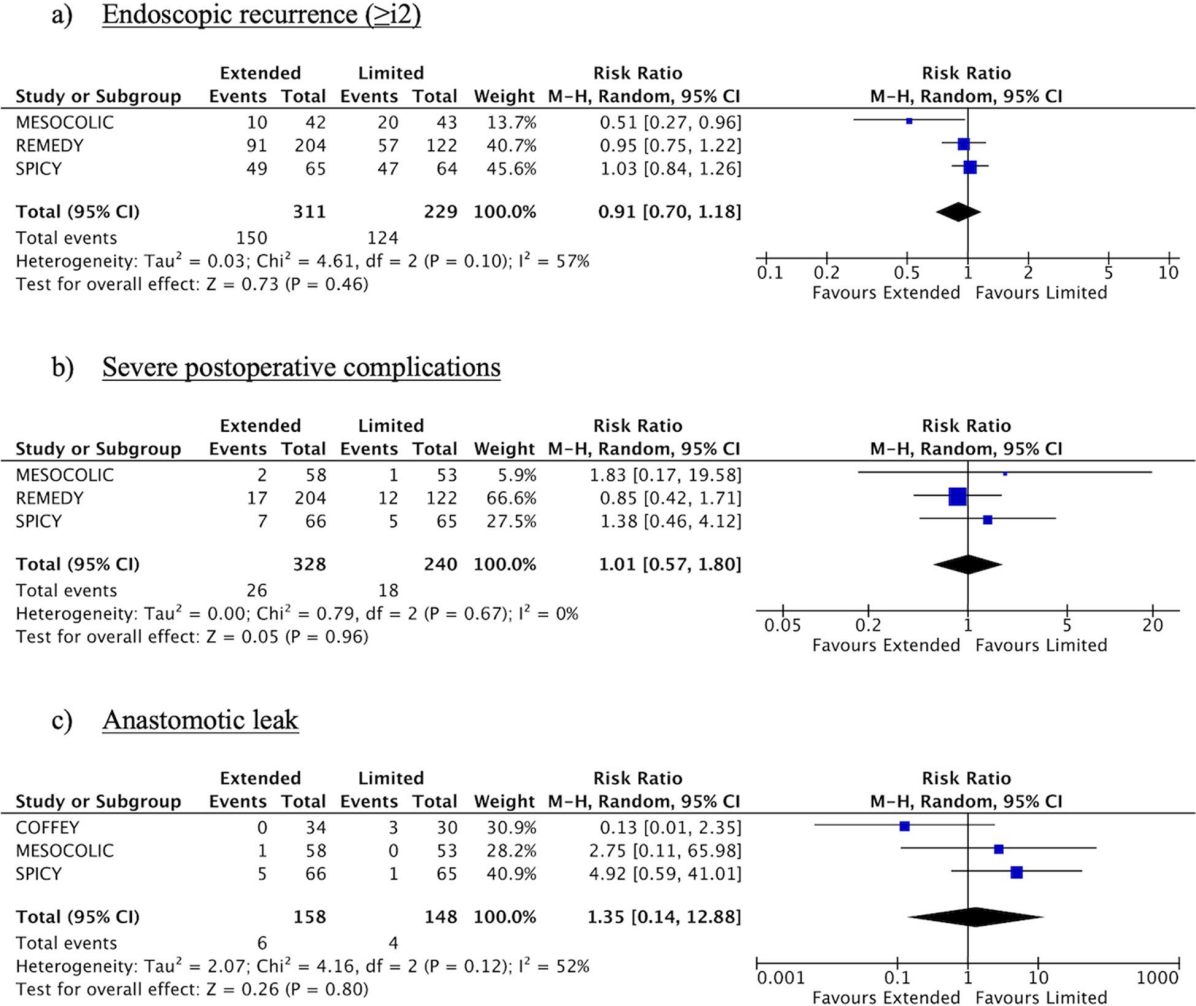
Forest plots

Three studies reported the rate of endoscopic recurrence after surgery according to a Rutgeerts score ≥ i2 [29, 30, 32]. Pooled data from these studies showed endoscopic recurrence in 48.2% of patients who underwent EME and in 54.1% of those who underwent LME; the overall RR was 0.91 (95%CI: 0.70–1.18; p = 0.46) with high heterogeneity (I^2^ = 57%) (Fig. 2). The process of data extraction revealed inconsistencies in the reporting of endoscopic recurrence in the Mesocolic trial [30]. To adopt the most conservative approach, we used the data reported in the publication but also performed a sensitivity analysis with corrected numbers (eFigure 2), resulting in minor changes in the overall findings: RR 0.92 (95%CI: 0.73–1.17; p = 0.51) with moderate heterogeneity (I^2^ = 48%).

### Secondary outcomes

No significant differences were found between the two types of resection in terms of moderate (≥ i2b, EME 32.7% vs. LME 39.2%; RR 0.80; 95%CI: 0.46–1.40; p = 0.43; I^2^ = 47%) or severe (≥ i3, EME 12.1% vs. LME 15%; RR 0.57; 95%CI: 0.09–3.77; p = 0.56; I^2^ = 68%) endoscopic recurrence. Anastomotic leak rates were 3.8% for EME vs. 2.6% for LME (RR: 1.35; 95% CI: 0.14–12.88; p = 0.80; I^2^ = 52%). Similarly, no significant group-related differences were observed regarding operative time, severe postoperative complications (Clavien–Dindo ≥ 3), or length of hospital stay (Fig. 2, eFigure 2). The SPICY trial was the only study reporting data on conversion to open surgery, with 0% for EME and 3% for LME (p = 0.24) [29].

Regarding surgical recurrence, two studies reported the incidence of additional CD-related surgical procedures at any intestinal location. Mineccia et al. reported 5-year time-to-event estimates for the entire cohort, indicating an overall recurrence rate of 4%. They also presented subgroup-specific rates, 2.8% for patients who underwent mesenteric resection and 4% for those with mesenteric preservation, compared using the log-rank test (p = 0.6). However, these figures are mathematically inconsistent unless subgroup sizes or event counts were either misreported or influenced by censoring in ways not clearly described in the publication. Coffey et al. reported surgical recurrence as a binary outcome: “One patient in Cohort B [2.9% of total cohort] required reoperation for a CD-related indication. Nine patients in Cohort A [30%] required reoperation for a CD-related indication (cumulative incidence 40% vs. 10%, log rank test p = 0.003)”. No data were provided regarding follow-up duration. In our view, any attempt to quantitatively synthesize these heterogeneous and incompletely reported outcomes would be methodologically flawed and potentially misleading. Accordingly, and in line with the guidance outlined in the Cochrane Handbook, as well as our commitment to transparency and methodological rigor, we chose not to include this outcome in the pooled analysis.

### Study quality assessment

The risk-of-bias assessment is shown in eFigure 3. One study was classified as being at low risk of bias [29], two studies [30, 32] at moderate risk of bias, and one study at high risk of bias [13]. By applying the GRADE system, the overall quality of the evidence from the present systematic review was rated as moderate to low.

### Registered studies

The systematic review found nine unpublished studies registered in www.clinicaltrials.gov and www.isrctn.com. NCT03172143 is an RCT started by the same research team as the SPICY trial, which was retired in 2019 after the inclusion of two participants. NCT02542904 is an RCT conducted in one of the centers that subsequently participated in the Mesocolic trial. The study was completed in September 2018, but no results have been published to date. NCT06550843 is a retrospective case–control study conducted in another center participating in the Mesocolic trial (Mesocolic study period: February 2019 to December 2022). The study compared a prospective sample of patients undergoing LME between January and August 2021 with a retrospective cohort of patients undergoing mesentery-guided resection (i.e., resection of the macroscopically affected mesenterium) between January 2013 and July 2020. The study was registered in August 2024, but no results were available at the end of the screening period. The search also revealed three ongoing multicenter RCTs, for which patients are currently being recruited (NCT04578392, NCT06241170, and ISRCTN16900055), one RCT not yet recruiting (NCT06324838), and two Canadian prospective cohort studies involving approximately 30 patients each, with an unknown status (NCT04266600 and NCT04539665).

## Discussion

The present systematic review and meta-analysis focusing on primary ileocolic resections for CD suggest that there is no significant difference in the risk of endoscopic recurrence between EME and LME. No significant group-related differences were also observed in the risk of anastomotic leak or other adverse perioperative events. However, the evidence is limited to four studies, of which only two were RCTs. Moreover, a non-negligible number of registered trials with unpublished results were found on this topic.

Our study represents the first meta-analysis to include only patients who underwent primary ileocolic resections for CD. In contrast, previous meta-analyses included a broader range of intestinal resections, which notably influenced the inclusion of the study by Abdulkarim et al., published in 2023 [33]. This retrospective analysis involved 3,709 patients who underwent various types of colectomies for CD, drawn from the American College of Surgeons National Surgical Quality Improvement Program (ACS-NSQIP) colectomy-specific database. In that study, the classification of mesenteric-sparing versus extensive resections was based solely on whether fewer than 12 or 12 or more lymph nodes were retrieved, a highly questionable decision that introduces significant bias. This source of bias affects all previous meta-analyses, as the study by Abdulkarim et al. contributed over 80% of the total patient population included in their analyses [14–17]. Moreover, each conducted a pooled analysis of surgical recurrence without adequately considering the differing nature and reporting of outcomes across the included studies. This methodological oversight likely explains the divergent results obtained despite the inclusion of overlapping datasets.

The pioneering study by Coffey et al., published in 2018, was the first retrospective evaluation suggesting the clinical relevance of including the mesentery in ileocolic resections for CD [13]. The authors reported a significantly reduced CD-related reoperation rate (i.e., surgical recurrence) following mesenteric excision: 2.9% (1/34) in the EME group versus 30% (9/30) in the LME group. They also identified surgical technique as an independent determinant of recurrence but provided no data on intra- or postoperative outcomes. Apart from the small sample size, two major confounders warrant discussion. First, the LME group underwent surgery between January 2004 and April 2010, whereas the EME group underwent surgery after August 2010. Differences in follow-up duration and postoperative treatment protocols may have affected the results. Notably, the LIR!C trial, an open-label RCT conducted across 29 teaching hospitals and tertiary care centers in the UK and the Netherlands, compared surgical resection versus infliximab in patients with non-stricturing and immunomodulator-refractory ileocolic CD [34]. With a median follow-up of 63.5 months, 26% of the patients in the resection group, contemporary to the EME cohort of Coffey et al. (2008–2015), initiated anti-TNF therapy, and none required a second resection [35]. Lastly, a notable concern is the difference in inflammation of the ileal resection margins between the LME (79%) and EME (16%) groups. Given the well-established link between this finding and disease recurrence risk [7], its omission from univariate and multivariate analyses raises concerns regarding the study conclusions [36].

Years later, the SPICY study was the first published RCT assessing the impact of EME on postoperative endoscopic recurrence in 139 patients undergoing primary ileocolic resection for CD. The findings indicated that EME did not reduce endoscopic recurrence and may have been associated with a non-significant increased in postoperative complications [29]. Notably, SPICY was the only study included in this review in which the EME procedure preserved the ileocolic vessels; this could be a potential source of bias, as lymphatics course alongside the mesenteric vasculature. This technical modification was incorporated into the revised trial protocol following the withdrawal of an earlier trial (NCT03172143) that initially proposed high ligation of the feeding vessel at its origin.

The present work is also the first meta-analysis to include the Mesocolic trial, the second RCT published on this topic, which enrolled 116 patients randomized to either LME or EME, defined as resection up to 1 cm from the root of the ileocolic vessels. Patients underwent surgery between February 2019 and December 2022, and surgical recurrence was assessed as the primary outcome. The planned interim analysis suggested that EME significantly reduced endoscopic recurrence [30]. However, the COVID-19 pandemic affected the assessment of this outcome, leading to missing data in more than 20% of patients (23.42% overall; 25.86% in the EME group and 20.75% in the LME group). A critical review of the Mesocolic trial publication also revealed a discrepancy in the reported endoscopic recurrence rates. Specifically, the LME group had 20 events in the overall recurrence (Rutgeerts ≥ i2) calculation (text and Fig. 1B), yet only 19 events were attributable to this value based on the cumulative incidence of endoscopic recurrence and the distribution of Rutgeerts scores (text and Figs. 1C and 1D). Although this minor discrepancy did not alter the statistical significance of our meta-analysis, as demonstrated by the sensitivity analysis we performed, re-evaluating the published results with the corrected numerators resulted in a borderline statistical significance (p = 0.05) instead of the originally reported significant difference (p = 0.03) (eFigure 4). Finally, the coordinating center of this most recent RCT simultaneously conducted a monocenter RCT from January 2020 to July 2022, in which 81 patients with CD were randomized to receive either azathioprine (AZA) alone or AZA in combination with a three-month course of exclusive enteral nutrition (EEN) following intestinal resection [37]. Of the 81 patients included, 69% underwent ileocolic anastomosis. Within this subgroup, the endoscopic recurrence rate was significantly lower in the AZA + EEN group compared with the AZA-only group (29% [8/28] vs. 65% [17/26], p = 0.0007). The same center also conducted an overlapping pilot RCT between August 2016 and October 2021 involving 100 patients undergoing elective intestinal resection for CD to evaluate the role of prophylactic drainage [38]. The potential implications of the concurrent conduct of these trials were not disclosed in the available reports [30, 37, 38].

Specific considerations should be made for the included retrospective studies. In particular, data related to surgical recurrence were based solely on retrospective studies, which did not restrict the outcome to further surgeries in the ileocolic area but considered all CD-related reinterventions. It must be considered that the decision to pursue further surgery for clinical recurrence is influenced by several factors, such as the patient’s and surgeon’s preferences, the availability of non-surgical interventions (e.g., balloon dilation), and the effectiveness of medical treatments. Endoscopic recurrence assessment as a primary outcome is also prone to bias. A recent meta-analysis highlighted significant inter-observer variability in the assessment of endoscopic recurrence rates after ileocolic resection for CD, suggesting a high likelihood of misclassification [39]. Another recent meta-analysis based on individual patient data found no significant differences in clinical or surgical recurrence between Rutgeerts i2a and i2b categories, advocating a unified treatment strategy [40]. Regarding the two included RCTs, it must be considered that they reported data from the first endoscopic evaluation post-surgery (6–18 months) [29], whereas up to 40% of endoscopic recurrences may manifest beyond this initial postoperative assessment [41].

A novel approach, the antimesenteric, functional, end-to-end, handsewn ileocolic anastomosis (Kono-S), which excludes the mesentery from the staple line, has recently been described. Preliminary findings indicate a significant reduction in endoscopic recurrence scores and surgical recurrence rates [30, 42]. The SuPREMe-CD Study, which randomized 79 patients with ileocolic CD to receive either a Kono-S anastomosis or a conventional side-to-side anastomosis following LME, demonstrated a significant reduction in postoperative endoscopic and clinical recurrence rates [43]. Conversely, preliminary results from another RCT (NCT03256240) comparing the Kono-S anastomosis with the side-to-side functional end anastomosis in patients with CD undergoing ileocolic resection showed no significant difference between the two techniques in terms of endoscopic recurrence rates at 12–18 months. Clinical disease activity and CD subtype were identified as significant predictors of recurrence independent of anastomotic technique [44].

The ongoing MErKAT study was designed to specifically evaluate the independent effects of both EME and Kono-S on ileocolic CD. This multicenter, 2× 2 factorial, superiority RCT aims to recruit 308 patients undergoing ileocolic resection for primary or recurrent CD. The trial will investigate four treatment combinations: (1) Kono-S + EME, (2) Kono-S + LME, (3) Standard anastomosis + EME, and (4) Standard anastomosis + LME [45, 46].

### Limitations

This systematic review includes a small number of studies and patients; thus, several limitations must be acknowledged. Due to the paucity and heterogeneous literature available, bias related to small sample size, retrospective study designs, and variations in surgical techniques, treatment protocols, and follow-up durations must be considered when interpreting the results. One important limitation is the heterogeneity in the technical definitions of the extent of EME across included studies. Notably, the SPICY trial preserved the ileocolic vessels, whereas other studies performed resections closer to the mesenteric root with high ligation of the ileocolic vessels. This variation in surgical technique may have contributed to the observed null result, as differences in vascular preservation could impact postoperative recurrence rates. Despite its limitations, this systematic review and meta-analysis possesses several strengths that enhance the reliability and clinical relevance of the findings. First, we adhered to a strict methodological framework to minimize the risk of selection bias. Additionally, we thoroughly assessed the included studies for potential sources of bias, ensuring a rigorous and transparent evaluation of the available evidence. Second, we restricted inclusion to studies exclusively reporting on primary ileocolic resections, avoiding the confounding effect of recurrent disease. This decision improves the homogeneity of the study population and enhances the applicability of our findings to clinical practice. Furthermore, by limiting the scope to ileocolic resections, we avoided the heterogeneity introduced by including other types of intestinal or colorectal resections, which may have different recurrence patterns, postoperative outcomes, and management strategies. This approach strengthens the internal validity of our findings and enhances their direct applicability to surgical practice in CD. Additionally, all studies included in our meta-analysis exclusively reported outcomes for ileocolic resections only. As such, there was no need to verify subgroup data or resolve inconsistencies in subgroup reporting, as no studies involved other intestinal locations. Moreover, we prioritized endoscopic recurrence as the primary outcome, assessed using the Rutgeerts score, which provides a standardized and objective measure of disease recurrence. As previously noted, this approach may be superior to clinical or surgical recurrence assessments, which are influenced by subjective decision-making, variations in treatment availability, and differences in patient management strategies. By focusing on an objective and quantifiable outcome, our study provides more robust and clinically relevant conclusions. Finally, we provide a critical appraisal of the registered trials that were interrupted, uncompleted, or are still ongoing. Overall, with unpublished data from nine trials, this finding highlights, on one side, the clinical relevance of the topic, and on the other side, the non-negligible risk of critical methodological issues and lack of full transparency that may skew the available evidence. The publication of the ongoing trials and future large-scale, well-designed RCTs with standardized methodologies is advocated to establish definitive recommendations on mesenteric excision in ileocolic resections for CD.

## Conclusion

The present systematic review and meta-analysis show that EME does not provide a significant advantage over LME in reducing endoscopic recurrence after primary ileocolic resection for CD. Until more robust evidence becomes available, these findings suggest that surgical teams who routinely perform LME for ileocolic CD can continue their current approach.

## Supplementary Information

Below is the link to the electronic supplementary material.

Supplementary file1 (PDF 4280 KB)

## Data Availability

All the data is available upon reasonable request.
