# Supplementary material for: Effect of extensive mesenteric excision on primary ileocolic resection outcomes in Crohn’s disease patients: a systematic review with meta-analysis
Source: Int J Colorectal Dis. 2025 Dec 2;40(1):243. doi: 10.1007/s00384-025-05043-0 (PMC12672786; doi:10.1007/s00384-025-05043-0)
Supplement: Supplementary file 1 — Supplementary file1 (PDF 4280 KB) [file 384_2025_5043_MOESM1_ESM.pdf]

Supplemental Material

Manuscript title: “Effect of extensive mesenteric excision on primary ileocecal resection outcomes in Crohn’s disease patients: a systematic review with meta-analysis.

eTable 1. PRISMA 2020 checklist
eTable 2. AMSTAR-2 checklist
eTable 3. Examples of searches
eTable 4. Summary of the studies included and excluded on the full-text analysis

eFigure 1. Funnel plots
eFigure 2. Forest plots
eFigure 3. Risk of bias assessment
eFigure 4. Chi-square calculation in MESOCOLIC trial

eTable 1. PRISMA 2020 checklist

Section and Topic	Item #	Checklist item	Location where item is reported
**TITLE**			
Title	1	Identify the report as a systematic review.	1
**ABSTRACT**			
Abstract	2	See the PRISMA 2020 for Abstracts checklist.	2-3
**INTRODUCTION**			
Rationale	3	Describe the rationale for the review in the context of existing knowledge.	4-5
Objectives	4	Provide an explicit statement of the objective(s) or question(s) the review addresses.	5
**METHODS**			
Eligibility criteria	5	Specify the inclusion and exclusion criteria for the review and how studies were grouped for the syntheses.	5-6
Information sources	6	Specify all databases, registers, websites, organisations, reference lists and other sources searched or consulted to identify studies. Specify the date when each source was last searched or consulted.	6-7
Search strategy	7	Present the full search strategies for all databases, registers and websites, including any filters and limits used.	6-7, eTable 3
Selection process	8	Specify the methods used to decide whether a study met the inclusion criteria of the review, including how many reviewers screened each record and each report retrieved, whether they worked independently, and if applicable, details of automation tools used in the process.	7, eTable 4
Data collection process	9	Specify the methods used to collect data from reports, including how many reviewers collected data from each report, whether they worked independently, any processes for obtaining or confirming data from study investigators, and if applicable, details of automation tools used in the process.	7-8
Data items	10a	List and define all outcomes for which data were sought. Specify whether all results that were compatible with each outcome domain in each study were sought (e.g. for all measures, time points, analyses), and if not, the methods used to decide which results to collect.	7-8
	10b	List and define all other variables for which data were sought (e.g. participant and intervention characteristics, funding sources). Describe any assumptions made about any missing or unclear information.	7-8
Study risk of bias assessment	11	Specify the methods used to assess risk of bias in the included studies, including details of the tool(s) used, how many reviewers assessed each study and whether they worked independently, and if applicable, details of automation tools used in the process.	8
Effect measures	12	Specify for each outcome the effect measure(s) (e.g. risk ratio, mean difference) used in the synthesis or presentation of results.	9
Synthesis	13a	Describe the processes used to decide which studies were eligible for each synthesis (e.g. tabulating the study intervention	6-7-8

Section and Topic	Item #	Checklist item	Location where item is reported
methods		characteristics and comparing against the planned groups for each synthesis (item #5)).	
	13b	Describe any methods required to prepare the data for presentation or synthesis, such as handling of missing summary statistics, or data conversions.	7-8
	13c	Describe any methods used to tabulate or visually display results of individual studies and syntheses.	6-7-8
	13d	Describe any methods used to synthesize results and provide a rationale for the choice(s). If meta-analysis was performed, describe the model(s), method(s) to identify the presence and extent of statistical heterogeneity, and software package(s) used.	6-7-8-9
	13e	Describe any methods used to explore possible causes of heterogeneity among study results (e.g. subgroup analysis, meta-regression).	9
	13f	Describe any sensitivity analyses conducted to assess robustness of the synthesized results.	10
Reporting bias assessment	14	Describe any methods used to assess risk of bias due to missing results in a synthesis (arising from reporting biases).	Not applicable
Certainty assessment	15	Describe any methods used to assess certainty (or confidence) in the body of evidence for an outcome.	8-9 eFigure 1-2
**RESULTS**			
Study selection	16a	Describe the results of the search and selection process, from the number of records identified in the search to the number of studies included in the review, ideally using a flow diagram.	9, Figure 1
	16b	Cite studies that might appear to meet the inclusion criteria, but which were excluded, and explain why they were excluded.	9, eTable 4
Study characteristics	17	Cite each included study and present its characteristics.	9-10, Table 1, Table 2
Risk of bias in studies	18	Present assessments of risk of bias for each included study.	11, eFigure 3
Results of individual studies	19	For all outcomes, present, for each study: (a) summary statistics for each group (where appropriate) and (b) an effect estimate and its precision (e.g. confidence/credible interval), ideally using structured tables or plots.	10, 11, Table 1, Table 2, Figure 2, eFigure 2
Results of syntheses	20a	For each synthesis, briefly summarise the characteristics and risk of bias among contributing studies.	11, eFigure 3
	20b	Present results of all statistical syntheses conducted. If meta-analysis was done, present for each the summary estimate and its precision (e.g. confidence/credible interval) and measures of statistical heterogeneity. If comparing groups, describe the direction of the effect.	10, 11, Figure 2, eFigure 2

Section and Topic	Item #	Checklist item	Location where item is reported
	20c	Present results of all investigations of possible causes of heterogeneity among study results.	10, 11, Figure 2, eFigure 2
	20d	Present results of all sensitivity analyses conducted to assess the robustness of the synthesized results.	13, eFigure 4
Reporting biases	21	Present assessments of risk of bias due to missing results (arising from reporting biases) for each synthesis assessed.	Not applicable
Certainty of evidence	22	Present assessments of certainty (or confidence) in the body of evidence for each outcome assessed.	10, 11, Figure 2, eFigure 1
**DISCUSSION**			
Discussion	23a	Provide a general interpretation of the results in the context of other evidence.	12-13-14
	23b	Discuss any limitations of the evidence included in the review.	14-15
	23c	Discuss any limitations of the review processes used.	14-15
	23d	Discuss implications of the results for practice, policy, and future research.	12-13-14-15
**OTHER INFORMATION**			
Registration and protocol	24a	Provide registration information for the review, including register name and registration number, or state that the review was not registered.	5
	24b	Indicate where the review protocol can be accessed, or state that a protocol was not prepared.	PROSPERO
	24c	Describe and explain any amendments to information provided at registration or in the protocol.	None
Support	25	Describe sources of financial or non-financial support for the review, and the role of the funders or sponsors in the review.	1
Competing interests	26	Declare any competing interests of review authors.	1
Availability of data, code and other materials	27	Report which of the following are publicly available and where they can be found: template data collection forms; data extracted from included studies; data used for all analyses; analytic code; any other materials used in the review.	1

eTable 2

AMSTAR 2

1. Did the research questions and inclusion criteria for the review include the components of PICO?

For Yes:
- X Population
- X Intervention
- X Comparator group
- X Outcome

Optional (recommended)
- X Timeframe for follow-up

- X Yes
- ☐ No

2. Did the report of the review contain an explicit statement that the review methods were established prior to the conduct of the review and did the report justify any significant deviations from the protocol?

For Partial Yes:
The authors state that they had a written protocol or guide that included ALL the following:
- X review question(s)
- X a search strategy
- X inclusion/exclusion criteria
- X a risk of bias assessment

For Yes:
As for partial yes, plus the protocol should be registered and should also have specified:
- X a meta-analysis/synthesis plan, if appropriate, *and*
- X a plan for investigating causes of heterogeneity
- X justification for any deviations from the protocol

- X Yes
- ☐ Partial Yes
- ☐ No

3. Did the review authors explain their selection of the study designs for inclusion in the review?

For Yes, the review should satisfy ONE of the following:
- ☐ *Explanation for* including only RCTs
- ☐ OR *Explanation for* including only NRSI
- X OR *Explanation for* including both RCTs and NRSI

- X Yes
- ☐ No

4. Did the review authors use a comprehensive literature search strategy?

For Partial Yes (all the following):
- X searched at least 2 databases (relevant to research question)
- X provided key word and/or search strategy
- X justified publication restrictions (eg, language)

For Yes, should also have (all the following):
- X searched the reference lists/bibliographies of included studies
- X searched trial/study registries
- X included/consulted content experts in the field
- X where relevant, searched for grey literature
- X conducted search within 24 months of completion of the review

- X Yes
- ☐ Partial Yes
- ☐ No

5. Did the review authors perform study selection in duplicate?

For Yes, either ONE of the following:
- X at least two reviewers independently agreed on selection of eligible studies and achieved consensus on which studies to include
- ☐ OR two reviewers selected a sample of eligible studies and achieved good agreement (at least 80 per cent), with the remainder selected by one reviewer

- X Yes
- ☐ No

6. Did the review authors perform data extraction in duplicate?

For Yes, either ONE of the following:
- X at least two reviewers achieved consensus on which data to extract

- X Yes

from included studies<br>☐ OR two reviewers extracted data from a sample of eligible studies and achieved good agreement (at least 80 per cent), with the remainder extracted by one reviewer		☐ No

7. Did the review authors provide a list of excluded studies and justify the exclusions?

For Partial Yes:	For Yes, must also have:	
X provided a list of all potentially relevant studies that were read in full text form but excluded from the review	X Justified the exclusion from the review of each potentially relevant study	X Yes<br>☐ Partial Yes<br>☐ No

8. Did the review authors describe the included studies in adequate detail?

For Partial Yes (ALL the following):	For Yes, should also have ALL the following:	
X described populations<br>X described interventions<br>X described comparators<br>X described outcomes<br>X described research designs	X described population in detail<br>X described intervention and comparator in detail (including doses where relevant)<br>X described study's setting<br>X timeframe for follow-up	X Yes<br>☐ Partial Yes<br>☐ No

9. Did the review authors use a satisfactory technique for assessing the risk of bias (RoB) in individual studies that were included in the review?

**RCTs**<br>For Partial Yes, must have assessed RoB from	For Yes, must also have assessed RoB from:	
X unconcealed allocation, *and*<br>X lack of blinding of patients and assessors when assessing outcomes (unnecessary for objective outcomes such as all cause mortality)	X allocation sequence that was not truly random, *and*<br>X selection of the reported result from among multiple measurements or analyses of a specified outcome	X Yes<br>☐ Partial Yes<br>☐ No<br>☐ Includes only NRSI
**NRSI**<br>For Partial Yes, must have assessed RoB:	For Yes, must also have assessed RoB:	
X from confounding, *and*<br>X from selection bias	X methods used to ascertain exposures and outcomes, *and*<br>X selection of the reported result from among multiple measurements or analyses of a specified outcome	X Yes<br>☐ Partial Yes<br>☐ No<br>☐ Includes only RCTs

10. Did the review authors report on the sources of funding for the studies included in the review?

For Yes	
X Must have reported on the sources of funding for individual studies included in the review. Note: Reporting that the reviewers looked for this information but it was not reported by study authors also qualifies	X Yes<br>☐ No

11. If meta-analysis was performed did the review authors use appropriate methods for statistical combination of results?

**RCTs**<br>For Yes:	
X The authors justified combining the data in a meta-analysis<br>X AND they used an appropriate weighted technique to combine study results and adjusted for heterogeneity if present	X Yes<br>☐ No<br>☐ No meta-analysis

X AND investigated the causes of any heterogeneity	conducted
**For NRSI**<br>For Yes:<br>X The authors justified combining the data in a meta-analysis<br>X AND they used an appropriate weighted technique to combine study results, adjusting for heterogeneity if present<br>X AND they statistically combined effect estimates from NRSI that were adjusted for confounding, rather than combining raw data, or justified combining raw data when adjusted effect estimates were not available<br>X AND they reported separate summary estimates for RCTs and NRSI separately when both were included in the review	X Yes<br>☐ No<br>☐ No meta-analysis conducted

12. If meta-analysis was performed, did the review authors assess the potential impact of RoB in individual studies on the results of the meta-analysis or other evidence synthesis?

For Yes:<br>☐ included only low risk of bias RCTs<br>X OR, if the pooled estimate was based on RCTs and/or NRSI at variable RoB, the authors performed analyses to investigate possible impact of RoB on summary estimates of effect	X Yes<br>☐ No<br>☐ No meta-analysis conducted

13. Did the review authors account for RoB in individual studies when interpreting/discussing the results of the review?

For Yes:<br>☐ included only low risk of bias RCTs<br>X OR, if RCTs with moderate or high RoB, or NRSI were included the review provided a discussion of the likely impact of RoB on the results	X Yes<br>☐ No

14. Did the review authors provide a satisfactory explanation for, and discussion of, any heterogeneity observed in the results of the review?

For Yes:<br>☐ There was no significant heterogeneity in the results<br>X OR if heterogeneity was present the authors performed an investigation of sources of any heterogeneity in the results and discussed the impact of this on the results of the review	X Yes<br>☐ No

15. If they performed quantitative synthesis did the review authors carry out an adequate investigation of publication bias (small study bias) and discuss its likely impact on the results of the review?

For Yes:<br>X performed graphical or statistical tests for publication bias and discussed the likelihood and magnitude of impact of publication bias	X Yes<br>☐ No<br>☐ No meta-analysis conducted

16. Did the review authors report any potential sources of conflict of interest, including any funding they received for conducting the review?

For Yes:<br>X The authors reported no competing interests OR<br>☐ The authors described their funding sources and how they managed potential conflicts of interest	X Yes<br>☐ No

eTable 3. Examples of searches

**PICOTS question:**<br>*(P) Participants*: Humans requiring primary ileocecal resection for Crohn's disease.<br>*(I) Intervention*: Extensive mesenteric excision.<br>*(C) Comparison:* Conventional limited mesenteric excision.<br>*(O) Outcome measures*:<br>- Primary outcome: Endoscopic (Rutgeerts scale ≥i2).<br>- Secondary outcomes: a) Severity of endoscopic recurrence Rutgeerts scale ≥i2b or ≥i3, b) surgical recurrence; c) operative time; d) conversion to open surgery; e) anastomotic leak; f) severe postoperative complications; e) length of hospital stay.<br>(T) Time: Short- and long-term.<br>(S) Setting: Inpatient and outpatient.
**Pubmed:**<br>#1 ("crohn disease/surgery"[MeSH Terms]) AND (humans[Filter])<br>#2 (((((((mesentery[MeSH Terms]) OR (Mesenter*[Text Word])) OR (mesenteric excision[Text Word])) OR (mesenteric sparing[Text Word])) OR (inflammatory bowel diseases[MeSH Terms])) OR (surgical procedures, operative[MeSH Terms])) OR (inflammatory bowel*[Text Word])) OR (general surgery[MeSH Terms])<br>#3 ((((Recurrence[MeSH Terms]) OR (Endoscopic recurrence[Text Word])) OR (clinical recurrence[Text Word])) OR (radiological recurrence[Text Word])) OR (ultrasonographic recurrence[Text Word])<br>#4 #1 AND #2 AND #3
**Embase:**<br>'colon crohn disease'/exp AND 'gastrointestinal surgery'/exp OR 'mesentery'/exp OR 'ileocolectomy'/exp OR 'mesentery excision':ti,ab,kw OR 'mesentery sparing':ti,ab,kw) AND 'recurrent disease'/exp OR 'clinic recurrence':ti,ab,kw OR 'radiological recurrence':ti,ab,kw OR 'ultrasonographic recurrence':ti,ab,kw OR 'endoscopic recurrence':ti,ab,kw
**Web of science:**<br>#1 TS=Crohn disease<br>#2 TS= (ileocolectomy OR mesenteric excision)<br>#3 TS= Recurrent disease<br>#4 #1 AND #2 AND #3
**Clinicaltrials:**<br>Condition: Crohn's disease / Other terms: Mesentery
**ISRCTN:**<br>Condition: Crohn's disease / text search: Mesenteric

eTable 4. Summary of the studies included and excluded on the full-text analysis.

Author (year)	Title	Inclusion / Exclusion	Reason for the exclusion
Abdulkarim (2023)	Extended versus limited mesenteric excision for operative Crohn's disease: 30-Day outcomes from the ACS-NSQIP database.	Excluded	Includes right colectomy and other segmental colectomies
Coffey (2018)	Inclusion of the Mesentery in Ileocolic Resection for Crohn's Disease is Associated With Reduced Surgical Recurrence.	Included	
Duan (2024)	Azathioprine Plus Exclusive Enteral Nutrition Versus Azathioprine Monotherapy for the Prevention of Postoperative Recurrence in Patients with Crohn's Disease: An Open-Label, Single-Centre, Randomized Controlled Trial.	Excluded	Does not provide data on EME vs. LME comparison
Duan (2025)	Postoperative Endoscopic Outcomes in the MESOCOLIC Trial Investigating Mesenteric-Based Surgery for Crohn's Disease.	Included	
Ewe (1989)	Postoperative recurrence of Crohn's disease in relation to radicality of operation and sulfasalazine prophylaxis: a multicenter trial.	Excluded	Does not provide data on EME vs. LME comparison
Holubar (2022)	Mesenteric Excision and Exclusion for Ileocolic Crohn's Disease: Feasibility and Safety of an Innovative, Combined Surgical Approach With Extended Mesenteric Excision and Kono-S Anastomosis.	Excluded	Case series of without comparison
Holubar (2022)	Safety & feasibility of targeted mesenteric approaches with Kono-S anastomosis and extended mesenteric excision in ileocolic resection and anastomosis in Crohn's disease.	Excluded	Does not provide data on EME vs. LME comparison
Jones (2025)	Mesenteric excision and endoscopic recurrence in ileocaecal Crohn's Disease: a single centre experience.	Excluded	Case series of without comparison
Mineccia (2022)	Has the Removing of the Mesentery during Ileo-Colic Resection an Impact on Post-Operative Complications and Recurrence in Crohn's Disease? Results from the Resection of the Mesentery Study (Remedy).	Included	
Van Der Does De Willebois (2024)	Effect of mesenteric sparing or extended resection in primary ileocolic resection for Crohn's disease on postoperative endoscopic recurrence (SPICY): an international, randomised controlled trial.	Included	
Zhu (2021)	Role of Extended Mesenteric Excision in Postoperative Recurrence of Crohn's Colitis: A Single-Center Study.	Excluded	Includes all colorectal resections

eFigure 1. Funnel Plots.

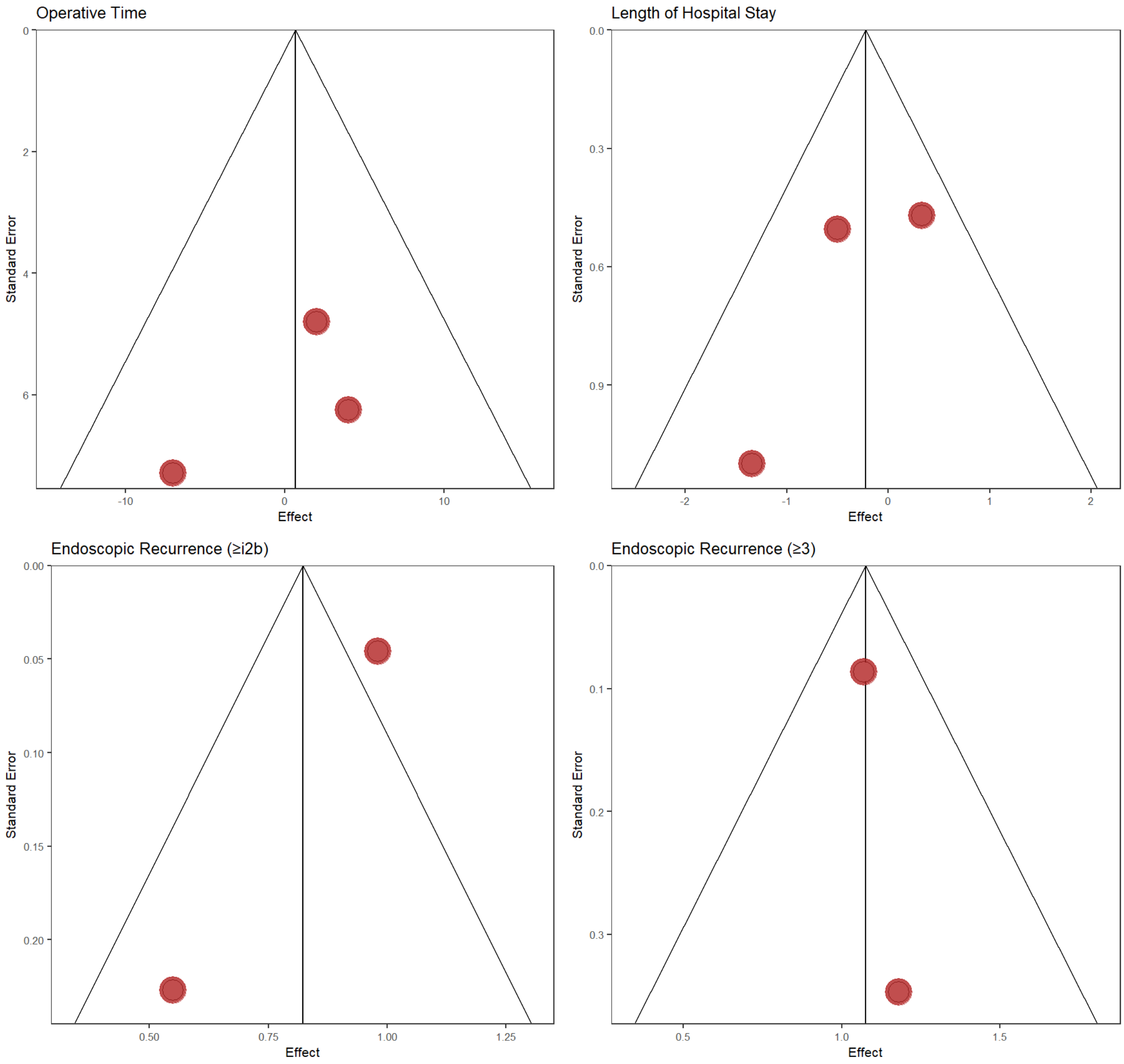

eFigure 2: Forest plots.

a) Operative time

Study or Subgroup	Extended Mean	Extended SD	Extended Total	Limited Mean	Limited SD	Limited Total	Weight	Mean Difference IV, Random, 95% CI
MESOCOLIC	147	23.57	58	145	26.67	53	49.5%	2.00 [−7.40, 11.40]
REMEDY	150	54	204	146	55	122	29.1%	4.00 [−8.25, 16.25]
SPICY	168	40.16	66	175	43.21	65	21.4%	−7.00 [−21.29, 7.29]
**Total (95% CI)**			**328**			**240**	**100.0%**	**0.66 [−5.96, 7.27]**

Heterogeneity: Tau² = 0.00; Chi² = 1.47, df = 2 (P = 0.48); I² = 0%
Test for overall effect: Z = 0.19 (P = 0.85)

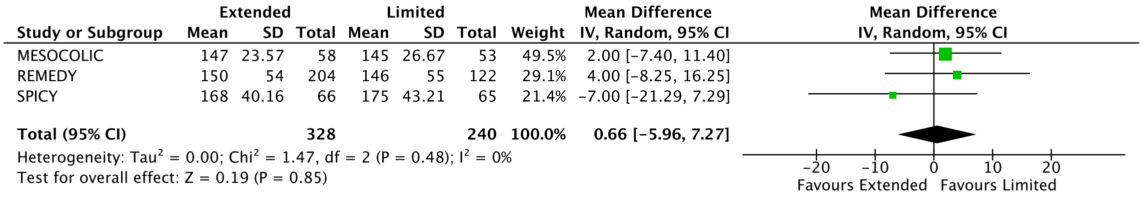

b) Length of hospital stay

Study or Subgroup	Extended Mean	Extended SD	Extended Total	Limited Mean	Limited SD	Limited Total	Weight	Mean Difference IV, Random, 95% CI
MESOCOLIC	8.66	3.04	58	10	5.33	53	20.4%	−1.34 [−2.97, 0.29]
REMEDY	8.5	5	204	9	4	122	38.4%	−0.50 [−1.49, 0.49]
SPICY	5.33	2.27	66	5	3.03	65	41.2%	0.33 [−0.59, 1.25]
**Total (95% CI)**			**328**			**240**	**100.0%**	**−0.33 [−1.19, 0.53]**

Heterogeneity: Tau² = 0.24; Chi² = 3.49, df = 2 (P = 0.17); I² = 43%
Test for overall effect: Z = 0.75 (P = 0.45)

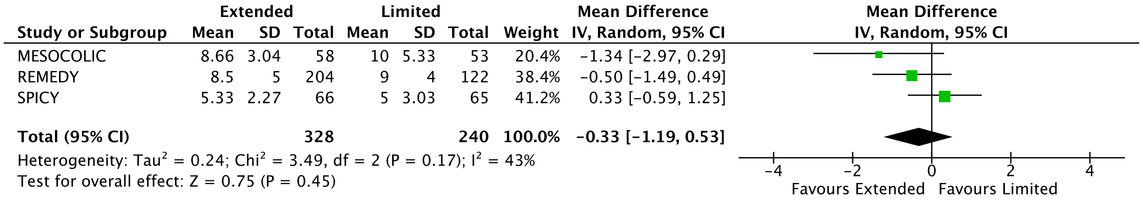

c) Endoscopic recurrence (≥i2b)

Study or Subgroup	Extended Events	Extended Total	Limited Events	Limited Total	Weight	Risk Ratio M-H, Random, 95% CI
MESOCOLIC	8	42	15	43	35.7%	0.55 [0.26, 1.15]
SPICY	27	65	27	64	64.3%	0.98 [0.66, 1.48]
**Total (95% CI)**		**107**		**107**	**100.0%**	**0.80 [0.46, 1.40]**
Total events	35		42			

Heterogeneity: Tau² = 0.08; Chi² = 1.89, df = 1 (P = 0.17); I² = 47%
Test for overall effect: Z = 0.79 (P = 0.43)

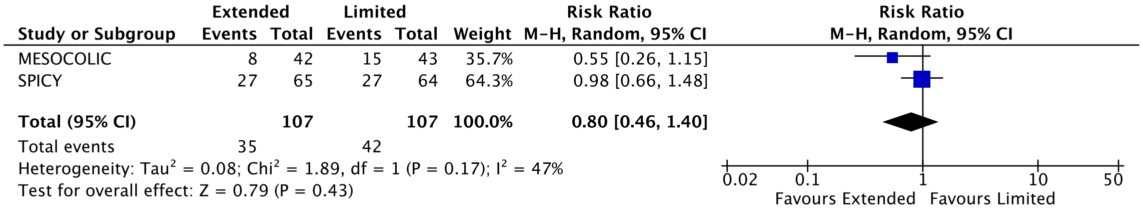

d) Endoscopic recurrence (≥3)

Study or Subgroup	Extended Events	Extended Total	Limited Events	Limited Total	Weight	Risk Ratio M-H, Random, 95% CI
MESOCOLIC	1	42	6	43	37.8%	0.17 [0.02, 1.36]
SPICY	12	65	10	64	62.2%	1.18 [0.55, 2.54]
**Total (95% CI)**		**107**		**107**	**100.0%**	**0.57 [0.09, 3.77]**
Total events	13		16			

Heterogeneity: Tau² = 1.34; Chi² = 3.11, df = 1 (P = 0.08); I² = 68%
Test for overall effect: Z = 0.59 (P = 0.56)

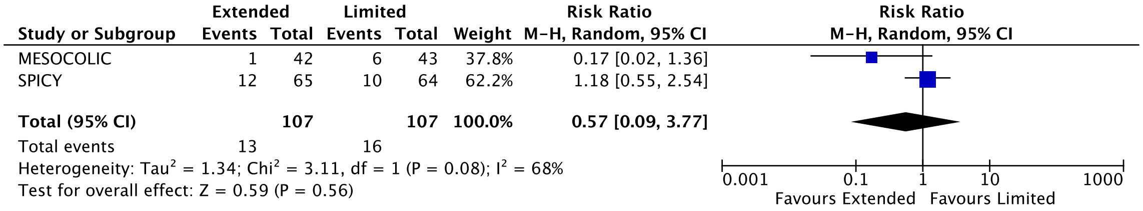

e) Sensitivity analysis of endoscopic recurrence (≥i2)

Study or Subgroup	Extended Events	Extended Total	Limited Events	Limited Total	Weight	Risk Ratio M-H, Random, 95% CI
MESOCOLIC	10	42	19	43	11.8%	0.54 [0.29, 1.02]
REMEDY	91	204	57	122	41.0%	0.95 [0.75, 1.22]
SPICY	49	65	47	64	47.2%	1.03 [0.84, 1.26]
**Total (95% CI)**		**311**		**229**	**100.0%**	**0.92 [0.73, 1.17]**
Total events	150		123			

Heterogeneity: Tau² = 0.02; Chi² = 3.88, df = 2 (P = 0.14); I² = 48%
Test for overall effect: Z = 0.65 (P = 0.51)

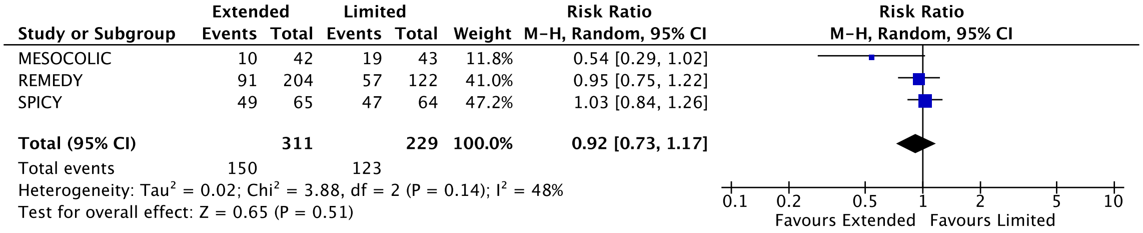

eFigure 3. Risk of bias assessment

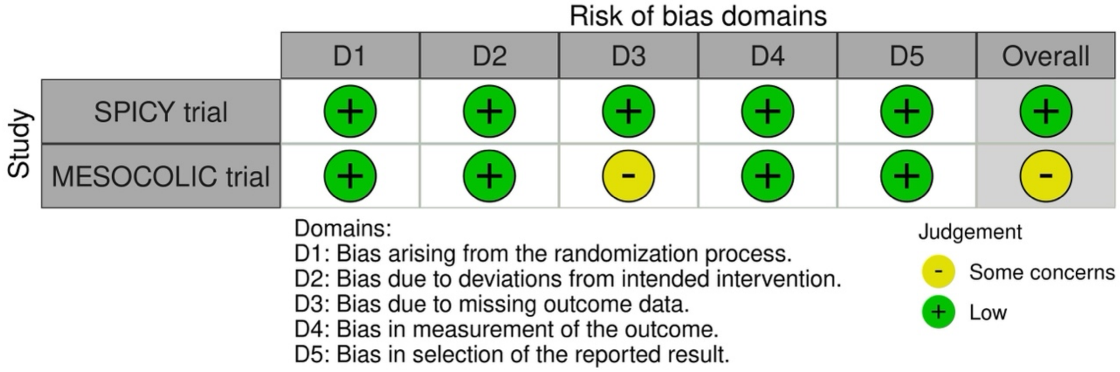

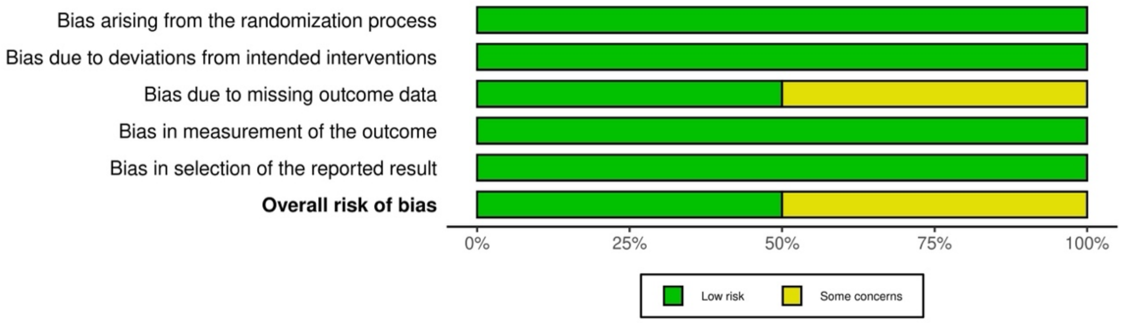

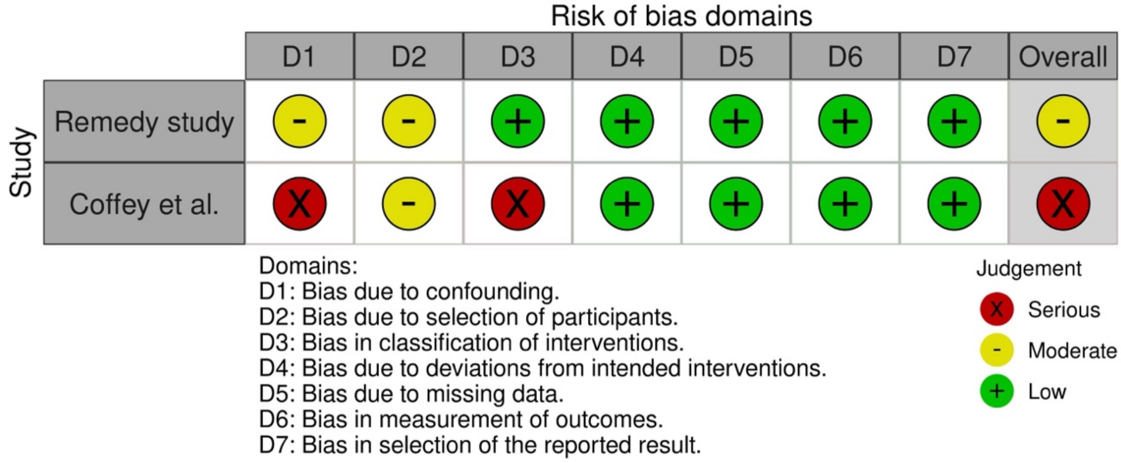

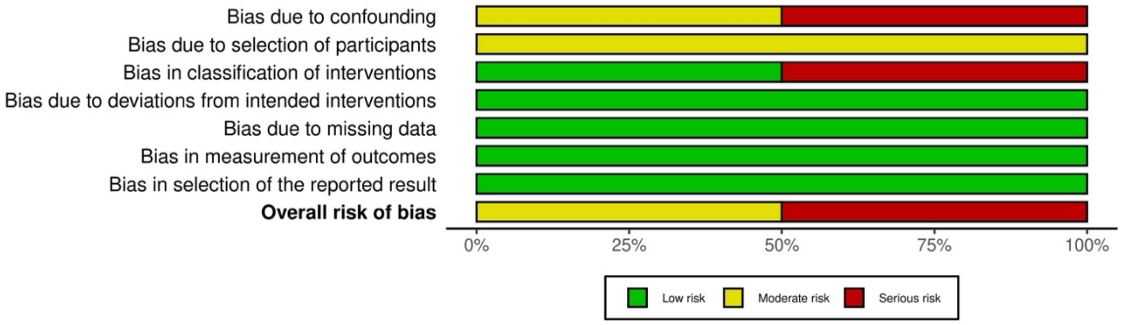

eFigure 4. Chi-square calculation in MESOCOLIC trial

Reported Data for Scenario 1 (Figure 1B and text):

- **EME**:
  - **ER Yes (≥i2)**: 10.
  - **ER No (i0, i1)**: 32.
  - **Total Patients**: 42.
- **LME**:
  - **ER Yes (≥i2)**: 20.
  - **ER No (i0, i1)**: 23.
  - **Total Patients**: 43.

Corrected Data for Scenario 2 (Figure 1C-D and text):

- **EME**:
  - **ER Yes (≥i2)**: 10.
  - **ER No (i0, i1)**: 32.
  - **Total Patients**: 42.
- **LME**:
  - **ER Yes (≥i2)**: 19.
  - **ER No (i0, i1)**: 24.
  - **Total Patients**: 43.

Observed Counts for Both Scenarios:

Scenario 1 (Figure 1B):

Group	ER Yes (≥i2)	ER No (i0, i1)	Total Patients
**EME**	10	32	42
**LME**	20	23	43

Scenario 2 (Figure 1C-D, Corrected):

Group	ER Yes (≥i2)	ER No (i0, i1)	Total Patients
**EME**	10	32	42
**LME**	19	24	43

Chi-squared Formula:

The Chi-squared (χ2χ2) test formula is: χ2=∑(O−E)2Eχ2=∑E(O−E)2

Where:

- OO = Observed frequency.
- EE = Expected frequency, calculated as:

E=Row Total×Column TotalGrand TotalE=Grand TotalRow Total×Column Total

---

<u>**Step-by-Step Calculation**</u>

Scenario 1 (Figure 1B):

Group	ER Yes (O)	ER No (O)	Row Total
**EME**	10	32	42
**LME**	20	23	43
**Col Totals**	30	55	85

Expected Counts:

E=Row Total×Column TotalGrand TotalE=Grand TotalRow Total×Column Total

Group	ER Yes (E)	ER No (E)
**EME**	42×30/85=14.8242×30/85=14.82	42×55/85=27.1842×55/85=27.18
**LME**	43×30/85=15.1843×30/85=15.18	43×55/85=27.8243×55/85=27.82

Chi-squared Contribution:

χ2=∑(O−E)2Eχ2=∑E(O−E)2

For EME:

χER Yes2=(10−14.82)214.82=1.56, χER No2=(32−27.18)227.18=0.85χER Yes2=14.82(10−14.82)2=1.56, χER No2 =27.18(32−27.18)2=0.85

For LME:

χER Yes2=(20−15.18)215.18=1.56, χER No2=(23−27.82)227.82=0.84χER Yes2=15.18(20−15.18)2=1.56, χER No2 =27.82(23−27.82)2=0.84

Total:

χ2=1.56+0.85+1.56+0.84=4.81χ2=1.56+0.85+1.56+0.84=4.81

Scenario 2 (Figure 1D):

Group	ER Yes (O)	ER No (O)	Row Total
**EME**	10	32	42
**LME**	19	24	43
**Col Totals**	29	56	85

Expected Counts:

Group	ER Yes (E)	ER No (E)
**EME**	42×29/85=14.3342×29/85=14.33	42×56/85=27.6742×56/85=27.67
**LME**	43×29/85=14.6743×29/85=14.67	43×56/85=28.3343×56/85=28.33

Chi-squared Contribution: For EME:

$\chi ER\ Yes2=(10-14.33)214.33=1.31$, $\chi ER\ No2=(32-27.67)227.67=0.68\chi ER\ Yes2=14.33(10-14.33)2=1.31$, $\chi ER\ No2=27.67(32-27.67)2=0.68$

For LME:

$\chi ER\ Yes2=(19-14.67)214.67=1.27$, $\chi ER\ No2=(24-28.33)228.33=0.66\chi ER\ Yes2=14.67(19-14.67)2=1.27$, $\chi ER\ No2=28.33(24-28.33)2=0.66$

Total:

$\chi 2=1.31+0.68+1.27+0.66=3.92\chi 2=1.31+0.68+1.27+0.66=3.92$

Results:

- **Scenario 1 (Figure 1B): $\chi 2=4.81, p\approx 0.03\chi 2=4.81, p\approx 0.03$ (significant).**
- **Scenario 2 (Figure 1C-D): $\chi 2=3.92, p\approx 0.05\chi 2=3.92, p\approx 0.05$ (borderline significant).**
